# Anti-PRL-3 Monoclonal Antibody inhibits the Growth and Metastasis of colorectal adenocarcinoma

**DOI:** 10.7150/jca.81702

**Published:** 2023-08-21

**Authors:** Shuning Sun, Lin Meng, Xiaofang Xing, Ningning Li, Qian Song, Dongbo Qiao, Like Qu, Caiyun Liu, Guo An, Zhongwu Li, Chenchao Shou, Shenyi Lian

**Affiliations:** 1 Key Laboratory of Carcinogenesis and Translational Research (Ministry of Education/Beijing), Department of Biochemistry and Molecular Biology, Peking University Cancer Hospital & Institute, Beijing, China.; 2 Key Laboratory of Carcinogenesis and Translational Research (Ministry of Education/Beijing), Department of Gastrointestinal Translational Research, Peking University Cancer Hospital & Institute, Beijing, China.; 3 Key Laboratory of Carcinogenesis and Translational Research (Ministry of Education/Beijing), Department of Pathology, Peking University Cancer Hospital & Institute, Beijing, China.; 4 Zhejiang Cancer Hospital, Hangzhou Institute of Medicine (HIM), Chinese Academy of Sciences, Hangzhou, Zhejiang, China.; 5 Key Laboratory of Carcinogenesis and Translational Research (Ministry of Education/Beijing), Department of Laboratory Animal, Peking University Cancer Hospital & Institute, Beijing, China.

**Keywords:** PRL-3, monoclonal antibody, colon cancer, metastasis

## Abstract

**Background:** Colon cancer is the one of leading causes of cancer-related death. Chemotherapy, radiotherapy and immunotherapy will be the mainstream in inoperable advanced cancer in clinics. Precision treatment is still lack in colon cancer.

**Materials and Methods:** We developed a series of mAbs targeting PRL-3 through different types of immunogens. The binding domains of mAbs were identified through the ELISA and Western blotting experiments. The antitumor activity of mAbs was verified by cell proliferation, migration and invasion experiments. Xenograft subcutaneous and metastatic models and patient derived Xenograft (PDX) model were established.

**Results:** mAb 12G12 targeting 77-120AA exhibited inhibition in migration and invasion experiments. 12G12 inhibited the migration of multiple types of cancer cells, including colon cancer, gastric cancer, esophagus cancer, liver cancer, lung cancer and pancreatic cancer cells. 12G12 decreased the tumor growth and metastasis in Xenograft subcutaneous and metastatic tumor model, respectively. The antitumor activity of mAb 12G12 was also confirmed in PDX model of gastric cancer. PRL-3 interacted with Golgi protein TMED10. Knockdown of TMED10 expression attenuated the cell migration triggered by purified GST-PRL-3 protein.

**Conclusion:** Our results confirmed the antitumor activity of mAb 12G12 in colorectal adenocarcinoma and provided a new potential targeted therapy of colon cancer.

## Introduction

The strategies of non-surgical therapy of tumor have been moving from chemotherapy or radiation therapy to individual precision therapy including targeted antitumor drugs, small molecular inhibition 1. Multiple drugs targeting tyrosine kinases have been applied in lung cancer and improved the clinical outcomes of a significant portion of NSCLC patients during the last ten years 2. In colon cancer, KRAS, NRAS, BRAF and PIK3CA were the well-known driver mutations. Almost 50% colon cancer patients were reported bearing KRAS mutation. Due to the smooth surface of KRAS protein, it has been considerate as “undruggable”. AMG510 targeting KRAS^G12C^ was still under assessment in the solid tumor 3, 4. Therefore, high specific antibody-based therapy with minor side effect would be a hopeful direction in the gastrointestinal cancer.

Our group devoted into the exploration of the biological function of PRL-3 in gastrointestinal cancer for decades. PRL-3, one member of the PRLs family, has been identified as an oncogene promoting cell migration and invasion in the many solid tumors 5. Our previous studies show that PRL-3 expression was related with stage, lymph node metastasis and poorer prognosis in colon cancer, breast cancer and ovarian cancer 6-9. In the transgenic mouse model, PRL-3 also participated in the initial process of inflammation-malignant transitions in colon cancer 10. PRL-3 enhanced the self-renewal of colon cancer cells and inhibition of PRL-3 may reduce the distant metastasis 11. PRL-3 played a critical role in T cell acute lymphoblastic leukemia (T-ALL) initiation and progression through promoting leukemia cell proliferation and migration 12, 13. PRL-3 serves as a downstream gene of SMARCA2 in t (4;14) multiple myeloma and activation of PRL-3 maintained MYC gene expression. PFI-3 (a BET inhibitor) could dissociated SMARCA2-NSD2 complex from PRL-3 promoter and induced myeloma cell apoptosis 14. Therefore, small molecular compound targeting PRL-3 would be potential therapy for both solid tumors and hematological malignancies.

Recently, a humanized antibody, PRL-3-zumab, was analyzed in the treatment of gastric cancer and liver cancers 15, 16. PRL-3-zumab has been in phase 2 clinical trial in Singapore 17, it gave us more confidence to explore therapeutic potential of novel PRL-3 antibodies. In this study, we generated mAb 12G12 which exhibited powerful antitumor activity in the in vitro and in vivo experiments. mAb 12G12 could be a potential antitumor drug in colon cancer.

## Materials and methods

### Cell cultures

Colon cancer cell lines HCT116, LoVo, SW480, gastric cancer cell lines AGS, BGC823, MGC803, lung cancer cell lines A549, H1299, PG, esophageal cancer cell line EC9706, pancreatic cancer cell lines ASPC-1, PANC-1, SW1990, liver cancer cell lines BEL7402, HEPG2, SMMC7721 were purchased from American Type Culture Collection (ATCC, Manassas, VA, USA). Esophageal cancer cell KYSE30-lm3 and KYSE450-lm2 were gifted by Professor Zhihua Liu (Cancer Institute, Cancer Hospital, Chinese Academy of Medical Sciences). Primary human fibroblasts IMR90 was obtained from Cell Center, Chinese Academy of Medical Science.

HEPG2, PANC-1, SW1990, EC9706, KYSE30-lm3, KYSE450-lm2 and IMR90 were cultured in Dulbecco's modified Eagles medium (DMEM) (ThermoFisher Scientific, Inc.) with 10% fetal bovine serum (Biology industries). AGS, MGC803, BG823, HCT116, SW620, SW480, A549, H1299, PG, BEL7402 and SMMC7721 were cultured in RPMI-1640 medium (ThermoFisher Scientific, Inc.) with 10% fetal bovine serum.

### PRLs mAbs development

We developed PRLs mAbs using three different types of immunogen: purified prokaryotic expression protein, prokaryotic plasmid pVAX1-Igκ-PRL-3(K-P3) and peptide cross-linked with KLH (KLH, Keyhole Limpet Hemocyanin). Generation of prokaryotic proteins and procedures of plasmid immunization were reported previously 18. For the peptide immunization, we choose the non-homogenous sequence on PRL-3 (compare with PRL-1 and PRL-2) for the peptide design. The sequence of Peptide 1: PGKVVEDWLSLVKAKFCEAEFDQVHFQPLPPAVVKLSDALC-KLH (77-95AA). The sequence of Peptide 2: RLRFKDPHTHKTRCCVMEFDQVHFQPLPPAVVKLSDALC-KLH (108-124AA). Mixture of two peptide (50 μg of each) with complete Freund's adjuvant (Sigma, F5881) was subcutaneously injected into Balb/c-nude mice. Two booster immunizations of peptide mixture with incomplete Freund's adjuvant (Sigma, F-5506) were taken on 3 weeks later. Tail blood was obtained for the titer examination. Hybridoma clones was developed by fusion of SP2/0 myeloma cells with Balb/c-nude mouse spleen-derived B cells which had high titer.

### ELISA assay

1.0 μg/mL indicated antigen were immobilized on the 96-well plates in 0.1 M carbonated buffer (NaHCO_3_ 0.1 M, Na_2_CO_3_ 0.1 M, pH 9.0-9.5). After 5% non-fat milk blocking, 1.0 μg/mL anti-PRL antibodies were incubated at 4℃ overnight. After washing by 0.1% PBST for 3 times, plates were incubated with HRP-coupled secondary antibody at room temperature for 45 minutes. After adding the substrate (1, 2-Diaminobenzene), the plated was measured at 450nm absorbance.

### Expression of prokaryotic proteins and Western blotting

PRLs plasmids (pGEX4T1-GST-PRL-1, pGEX4T1-GST-PRL-2, pGEX4T1-GST-PRL-3, His-PRL-3) and truncated PRL-3 plasmids (pGEX4T1-GST-PRL-3-p77, pGEX4T1-GST-PRL-3-p162, pGEX4T1-GST-PRL-3-p48, pGEX4T1-GST-PRL-3-p69, pGEX4T1-GST-PRL-3-p95 and pGEX4T1-GST-PRL-3-p120) were produced. E. coli expressing different plasmids was cultured in 3 mL LB medium at 37℃ for 12 hours, then transferred to a 500 mL LB media with Ampicillin (final concentration 1.0 μg/mL) for 24 hours. LB media was diluted to appropriate absorbance (OD600 = 0.6-0.8) and cultured at 37℃ for 6-8 hours with 0.5 mM IPTG. Cells were homogenized in the bacterial lysis buffer (1 mM DTT, 1 mM PMSF, 1 μg/mL lysosome, 1% TritonX-100) on ice for 30 minutes, followed by ultrasonic lysis and centrifugation at 12 000 rpm for 15mins at 4℃. Bacterial supernatant was loaded on the 12% SDS-PAGE gel and detected by GST antibody and the indicated anti-PRLs primary antibodies. Commercial PRL-3 antibody (clone 318, sc-130355) were purchased from Santa Cruz.

### Cell proliferation, migration and invasion assays

Cell Counting Kit (CCK)-8 (C0037, Beyotime, Shanghai, China) were used for cell proliferation experiments. 3 ×10^4^ of indicated colon cancer cells were re-suspended in 100 μl complete medium, and seed in the 96-well plates in triplicate. At 0, 24, 48, 72, and 96 hours, the medium was discarded and 100 μl fresh medium containing 10 μl CCk-8 was added to each well. After 2 hours incubation at 37℃, the spectrophotometer (Thermo Fisher Scientific) was used to measure the absorbance of each well at OD450nm.

For transwell chamber assays, 200 μl of re-suspended cells (2×10^5^/ml for migration and 10×10^5^/ml for invasion) were seeded on the upper chamber of each transwell (Becton Dickinson, San Jose, CA, USA), and 800 μl medium containing 10% FBS was added to the lower chamber. Cells were cultured at 37℃ for 24 hour (for migration) or 48 hours (for invasion). The cells penetrated the upper chamber were fixed in cold methanol, stained in 0.1% crystal violet, and counted in at least 6 randomly selected field under microscope.

### Xenograft subcutaneous tumor model and xenograft metastatic model

Animal study was approved by the Biomedical Ethical Committee of Peking University Cancer Hospital & Institute (License Number: EAEC 2019-05) and performed along established institutional animal welfare guidelines concordant with the US guidelines (NIH Publication #85-23, revised in 1985).

The 8-week old Balb/c-nude mice were subcutaneously implanted with 2×10^6^ LoVo cells in 200 μL of the PBS suspension to establish the xenograft subcutaneous tumor model. mAb 12G12 or IgG (200 μg in 100 μL) were administrated intravenously twice a week for 4 weeks (11). Mice were all sacrificed on day 31. Tumor size was measured using a caliper and tumor volume were calculated using the formula T_vol_=1/2×larger diameter × (smaller diameter)^ 2^.

The 8-week-old Balb/c-nude mice were intravenously implanted with 5×10^5^ LoVo cells in 200 μL of PBS suspension to establish the lung metastasis model. Following this, 12G12 (400 μg in 200 μL) or PBS (200 μL) were administrated intravenously twice a week for 3 weeks. The weight of mice was monitored every 7 days. All the mice were sacrificed on day 60, and the lung tissues were filled with the Bouin's solution before the detachment 6.

### Patients-derived xenograft (PDX) model

Balb/c-nude mice (4-6 weeks old, body weight, 18-20 g) were maintained and treated in accordance with Biomedical Ethical Committee of Peking University Cancer Hospital & Institute. Gastric cancer tissues from three patients were removed under sterile conditions. Tumor tissues were washed three times with a phosphate buffered saline (PBS) solution containing 2% penicillin/streptomycin, and necrotic tissue was removed. For PDX passaging, the tumor tissue was cut into 2 mm × 2 mm × 1 mm pieces and inoculated subcutaneously on the right side of the dorsal front of mice. Sixteen mice were randomly divided into PBS group and 12G12 group. After three day of tumor inoculation, PBS or 12G12 were injected twice a week for 2-3 weeks. Tumors were harvested once they reached the endpoint (> 10 mm in the largest dimension). Tumors were dissected, weighed, and photographed. Tumor volume was calculated using the formula (tumor volume = length × width^2^/2).

### Immunohistochemistry

Tissue samples from xenograft tumors (including xenograft subcutaneous tumor model, xenograft metastatic model and PDX model) were fixed in 4% formaldehyde for 24 hours, processed to the paraffin, and sectioned. For immunohistochemistry, 5 μm sections were deparaffinized in xylene and hydrated in alcohol. Endogenous peroxidase activity was then blocked by incubation in 3% hydrogen peroxide-methanol for 10 minutes. For the antigen recovery, the section was heated in a citrate buffer (10 mM Sodium Citrate, pH 6.0) for 15 minutes. After blocking, the primary antibodies against caspase-3(1:1000) (Lot: #9662, Cell Signaling), cleaved caspase-3 (5A1E) (1:400) (Lot: #9664, Cell Signaling) was applied at 4℃ overnight. EnVision+ TM (Dako) was used as the secondary antibody. Antibody binding was visualized by a standard streptavidin immunoperoxidase reaction. These sections were counterstained with hematoxylin.

The score of immunohistochemistry was graded by histochemistry score (H-Score) system. Evaluation was carried out independently by two experienced pathologists. The H-score system was the combination of proportional and intensity of positive tumor cells. H score = ΣPi(i+1). Pi, the ratio of the positive staining cell in all the tumor cells; i, the staining intensity.

### Small interfering RNA (siRNA) synthesis and, vector construction and transfection

The full-length sequence of human TMED10 eukaryotic plasmid (pCDNA3.0-myc-TMED10) was synthesized by Sangon Biotech (Suzhou, China). The full-length sequence of human PRL-3 eukaryotic plasmid pEBG-GST-PRL-3 was constructed previously in our lab. The siRNA siTMED10-1# (GCUAGUGACUGGCGCGUACTT), siTMED10-2# (CCAUAUUCUCUACUCCAAATT), siTMED10-3# (UCUUCUACCUGCGACGCUUTT) and control siRNA (UUCUCCGAACGUGUCACGUTT) were synthesized by GenePharma (Shanghai, China). Plasmids and siRNA were transfected with Lipofectamine2000 (Invitrogen) into HCT116 cells according to the manufacturer's instructions.

### Co-immunoprecipitation and GST pull-down

HCT116 cells were transiently transfected with GST-PRL-3, myc-TMED10 and vector for 48 hours. Cells were lysed in buffer (50 mM Tris-HCl, pH 7.5, 150 mM NaCl, 1 mM EDTA and 0.5% NP-40) containing complete protease inhibitors (Roche, USA), and the lysates were used for GST pull-down with Glutathione Sepharose or immunoprecipitation with anti-myc antibody 10. The precipitants were subjected to Western blotting with specific antibodies. Visualization was achieved with chemiluminescence.

### Statically analysis

The SPSS (version 26) software package (SPSS Inc, Chicago, IL) was used for statistical analysis.

Qualitative data are expressed as the mean ± standard deviation. Difference between groups were compared by one-way ANOVA or Student's t-test or χ2 test. GraphPad Prism 6 software (GraphPad Software, San Diego, CA) was used for statistical figure or chart display. All experiments were repeated at least three times with consistent results. P-value < 0.05 was considered statistically significant.

## Results

### PRLs mAbs development and identification of the immunogenic target domains

**Figure 1:**
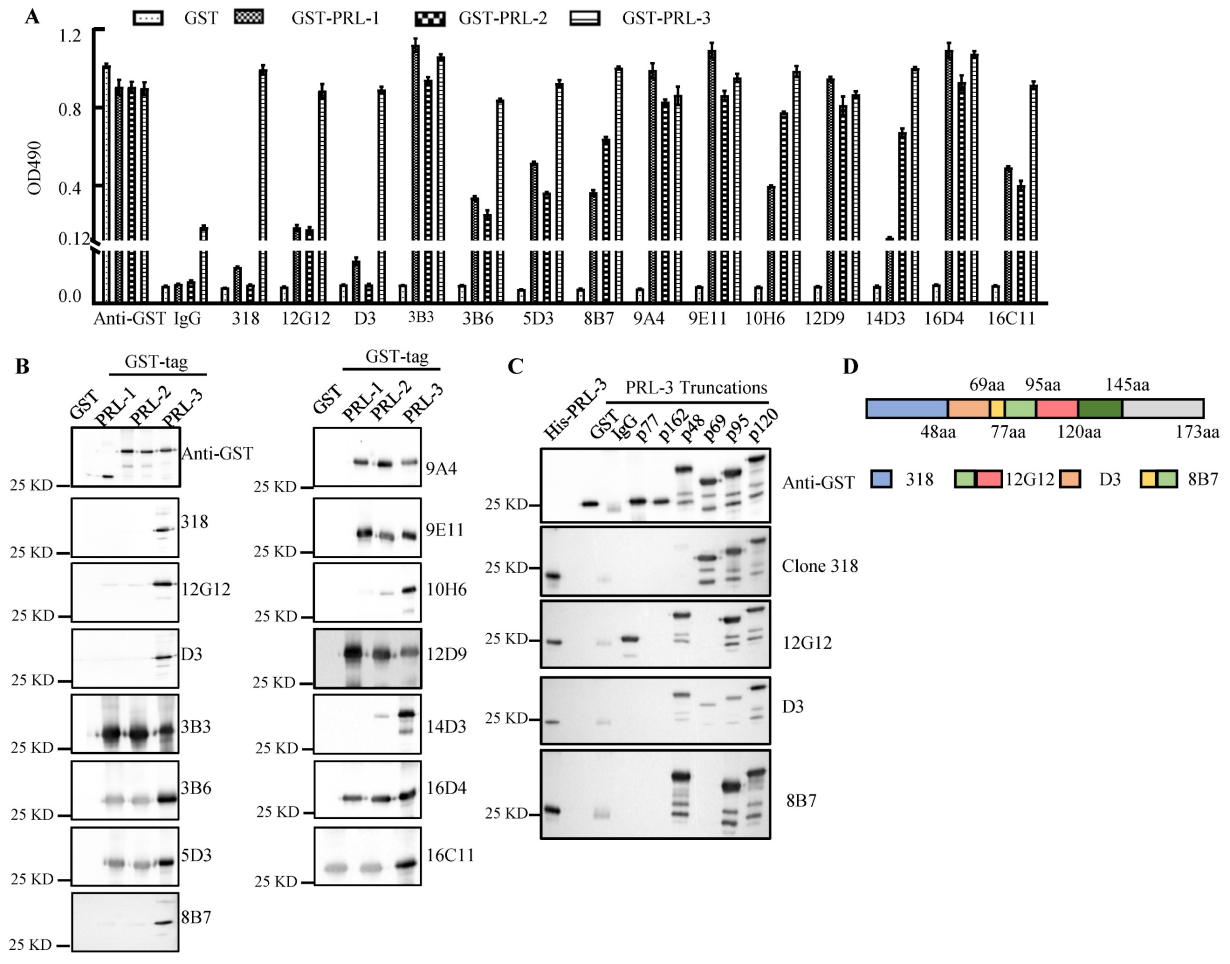
** Screening of anti-PRL-3 mAbs. A**. The binding affinity of anti-PRLs mAb to GST-PRLs proteins were measured by ELISA. **B**. The binding affinity of anti-PRLs mAb to GST-PRLs proteins were measured by Western blotting.** C.** The binding domains of specific anti-PRL-3 mAbs were determined by Western blotting with GST-tagged truncated PRL-3 proteins as antigens. *p*77, 77-98aa; *p*162, 162-173aa; *p* 48, 48-145aa; *p* 69, 1-69aa, *p* 95, 1-95aa, *p* 120, 1-120aa. **D.** Schematic of anti-PRL-3 mAbs and clone 318's binding domains.

A series of mAbs generated from 3 types of immunogen were quantified by the Coomassie brilliant blue staining using BSA protein as a standard (Supplemental Figure 1A). The mAbs (1.0 μg/ml) were used for ELISA and Western blotting of purified GST-PRLs proteins (Figure 1A and 1B). The molecular weight (MW) of each GST-PRL displayed on the blots (Figure 1B) was in accordance with the reported MWs of PRL-1 (22.1KDa), PRL-2 (17.4KDa) and PRL-3 (22.1KDa) 19. mAbs 12G12, D3, 8B7 and 10H6 were screened as candidates of specific anti-PRL-3 antibodies. To eliminate the cross-reaction of PRLs family, we loaded 10 times amounts of GST-PRL-1 or GST-PRL-2 (5.0 μg) with GST-PRL-3 (0.5 μg) (Supplementary Figure 1B). Except mAbs 10H6, which has little cross-reaction with 10 times GST-PRL-2 protein, other mAbs (12G12, D3 and 8B7) mostly reacted with GST-PRL-3, but not with 10 times GST-PRL-1 or GST-PRL-2 (Supplementary Figure 1B). Therefore, mAb 12G12 (peptide immunized), D3 (prokaryotic protein immunized) and 8B7 (prokaryotic plasmid immunized) were identified as specific antibodies against human PRL-3 (Figure 1A and 1B). To further confirm the binding domain of these mAbs on PRL-3 protein, we constructed 6 truncated GST-PRL-3 proteins with indicated amino acid deletions. The anti-PRL-3 mAbs (12G12, D3 and 8B7), commercial clone 318 (sc-130355) and GST antibody were used to detect these truncated proteins (Figure 1C). As summarized in Figure 1D, the potential binding domains of clone 318, D3 and 8B7 were 1-48AA, 48-69AA and 69-95 AA of PRL-3, respectively. 12G12 uniquely recognized 77-120 AA, which covering several phosphorylation sites of PRL-3^20^. Therefore, we supposed that mAb 12G12 may exhibit better biological activity than the others anti-PRL-3 mAbs.

### mAb 12G12 inhibited colon cancer cells migration and invasion

**Figure 2:**
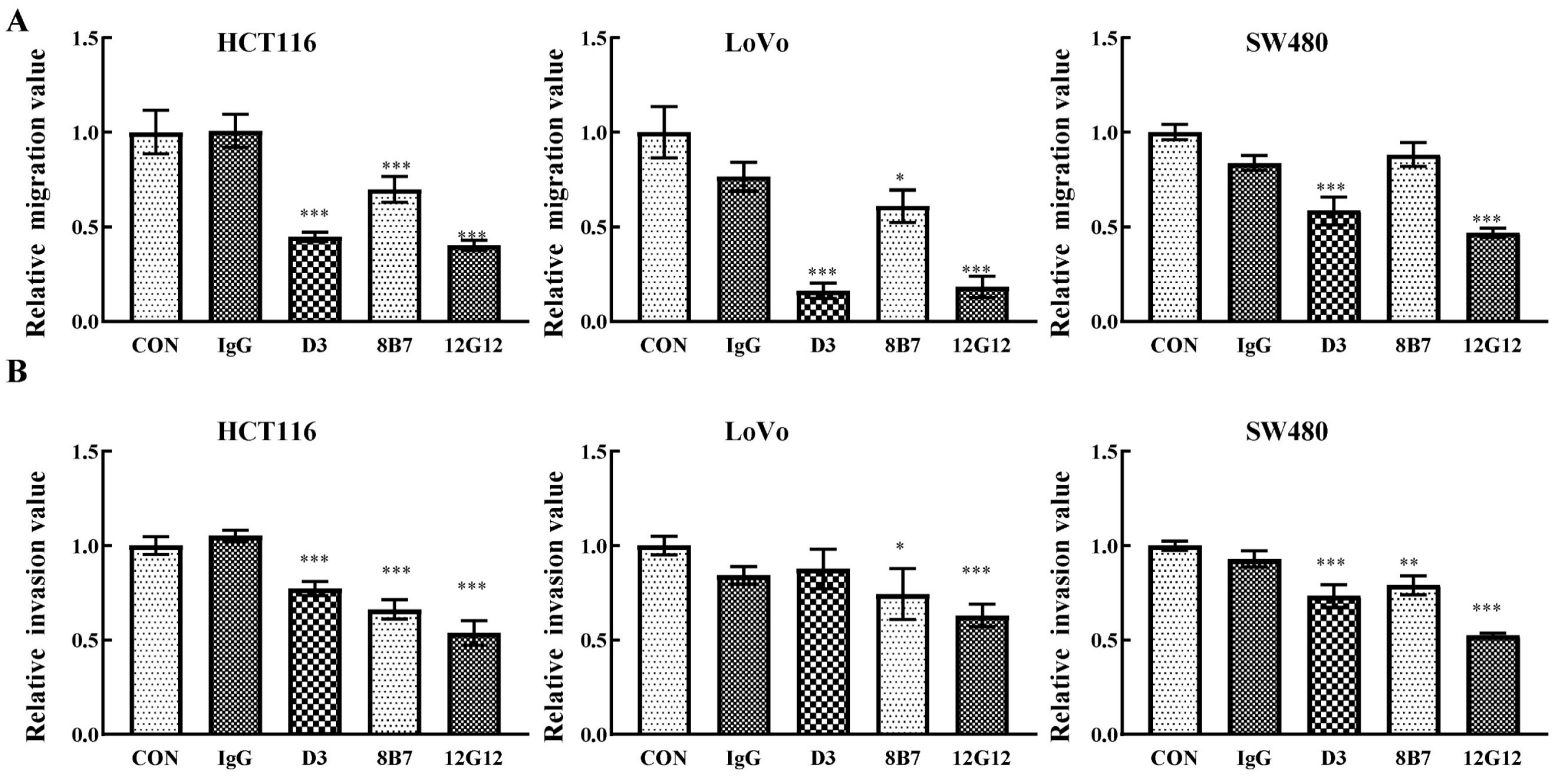
**mAb 12G12 inhibited colon cancer cells migration and invasion.** Transwell chamber migration assays **(A)** and Matrigel invasion assays** (B)** for HCT116, SW480 and LoVo cells treated with PBS, IgG (10.0 μg/ml) and 12G12 (2.0 μg/ml). The experiments were repeated three times. The values are the mean and standard deviation of normalize the number of each group with PBS. *, *p* <0.05; **, *p* <0.01; ***, *p* <0.001.

PRL-3 functions as an oncogene in the tumorigenesis and metastasis, and inhibition of PRL-3 by siRNA or inhibition of catalytic activity through mutation could decreased the motility of many solid cancer cells 5. We inspected the anti-metastatic ability of anti-PRL-3 mAbs. mAb 12G12 inhibited cell migration and invasion of HCT116 (inhibition rate: 40.3% ± 2.73%; 53.6% ± 6.4%, respectively), LoVo (inhibition rate: 18.3% ± 5.69%; 63.0% ± 5.91%, respectively) and SW480 cells (inhibition rate: 46.9% ± 2.41%; 52.3% ± 1.37%, respectively). Although D3 exhibited relatively higher sensitivity in ELISA and Western blotting (Figure 1A and 1B), it failed to inhibit invasion of LoVo cells. Moreover, 8B7 did not inhibit the cell migration of SW480 cells (Figure 2A and 2B). Thus, we chose mAb 12G12 in the subsequent analysis.

To extend the application of mAb 12G12, we detected the PRL-3 expression in human fibroblast IMR-90 and different types of cancer cell lines (Supplementary Figure 2A). PRL-3 generally expressed in these cancer cell lines with different levels, but not in IMR-90 cells. The expression of PRL-3 in esophagus cancer cell lines was lower than the other types of cancer cell lines. In the colon cancer cell lines, PRL-3 expressed lower in LoVo cell than in HCT116 and SW480 cell lines. This result was consistent with our previous study 10. In the gastric cancer cell lines, the expression of PRL-3 was higher in AGS and MGC823 cell lines. In the liver cancer cell lines, the PRL-3 expressed higher in SMMC 7721 than in BEL 7402 and HEPG2. In the lung cancer cell lines, the expression of PRL-3 in PG was lower than in A549 and H1299. In the pancreatic cancer cell lines, the PRL-3 expressed higher in SW1990 than in PANC-1 and ASPC-1. In the cell migration experiments, mAb 12G12 dramatically inhibited three colon cancer cell line (inhibition rate: HCT116, 62.2% ± 1.8%; LoVo, 36.4% ± 4.9%; SW480, 39.4% ± 4.6%) (Supplementary Figure 2B), three gastric cancer cell lines (inhibition rate: AGS, 28.0% ± 5.4% BGC823, 69.4% ± 5.9%; MGC 803, 52.5% ± 6.8%) (Supplementary Figure 2C), three esophagus cancer cell lines (inhibition rate: KYSE30-lm3, 49.8% ± 3.4%; KYSE450-lm2 74.0% ± 5.8%; EC9706, 65.8% ± 2.2%;) (Supplementary Figure 2D), TWO liver cancer cell lines (inhibition rate: HEPG2, 64.4% ± 4.3%; SMMC7721, 40.4% ± 4.3%) (Supplementary Figure 2E), two lung cancer cell lines (inhibition rate: H1299, 83.6% ± 7.5%; PG, 71.2% ± 7.7%) (Supplementary Figure 2F) and one pancreatic cancer cell line (inhibition rate:PANC-1, 54.9% ± 4.0%) (Supplementary Figure 2G). Together, we confirmed that mAb 12G12 exhibited general anti-migratory and anti-invasive ability on different cancer cell lines in vitro.

### mAb 12G12 inhibited colon cancer cell motility in a dose-dependent manner

**Figure 3:**
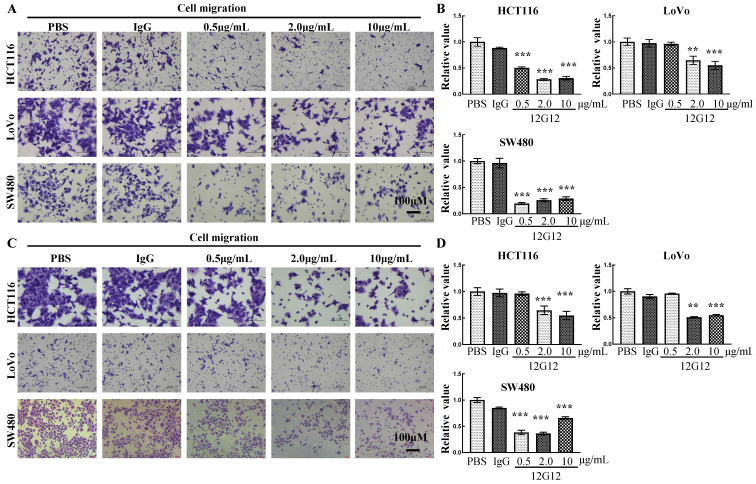
** mAb 12G12 inhibited colon cancer cell motility in a dose-dependent manner.** Transwell chamber migration assays **(A)** and Matrigel invasion assays **(B)** of HCT116, LoVo and SW480 cells with different 12G12 dosages (2.0 μg/ml, 5.0μg/ml,10.0 μg/ml). PBS and IgG (10.0μg/ml) treatment were set as blank and negative control. The experiments were repeated three times. The values are the mean and standard deviation of normalize the number of each group with PBS. *, *p* <0.05; **, *p* <0.01; ***, *p* <0.001.

To further verify the application of mAb 12G12, we utilized PBS (blank control), IgG (negative control) and different dose of 12G12 mAbs (0.5μg/ml, 2.0μg/ml and 10.0μg/ml) in the migration and invasion experiments. mAb 12G12 (2.0 μg/ml) significantly inhibited the migration and invasion of all these 3 colon cancer cells (migration inhibition rate: HCT116, 28.1% ± 2.0%; LoVo, 64.6% ± 7.92%; SW480, 19.5% ± 1.7%; invasion inhibition rate: HCT116, 51.0% ± 1.2%, LoVo, 55.5% ± 6.6%; SW480, 38.3% ± 4.4%) (Figure 3A and 3B). Loaded 12G12 to 10 μg/ml barely further increased the inhibition of cell migration or invasion in all these cell lines. 12G12 significantly inhibited proliferation of HCT116 and SW480 cells compared with PBS and IgG (Supplementary Figure 3A). However, we did not observe anti-proliferative ability of 12G12 in LoVo cells in three independent experiments. Our previous studies reported the dual effects of PRL-3 on the cancer cell proliferation, as both overexpression and knockdown of PRL-3 in colon cancer cells could inhibit cell proliferation through distinct mechanisms 10. These in vitro experiments indicated that mAb 12G12 may be utilized as a potential antitumor drug.

### mAb 12G12 inhibited colon cancer tumor growth and metastasis to Lung in mice model

**Figure 4:**
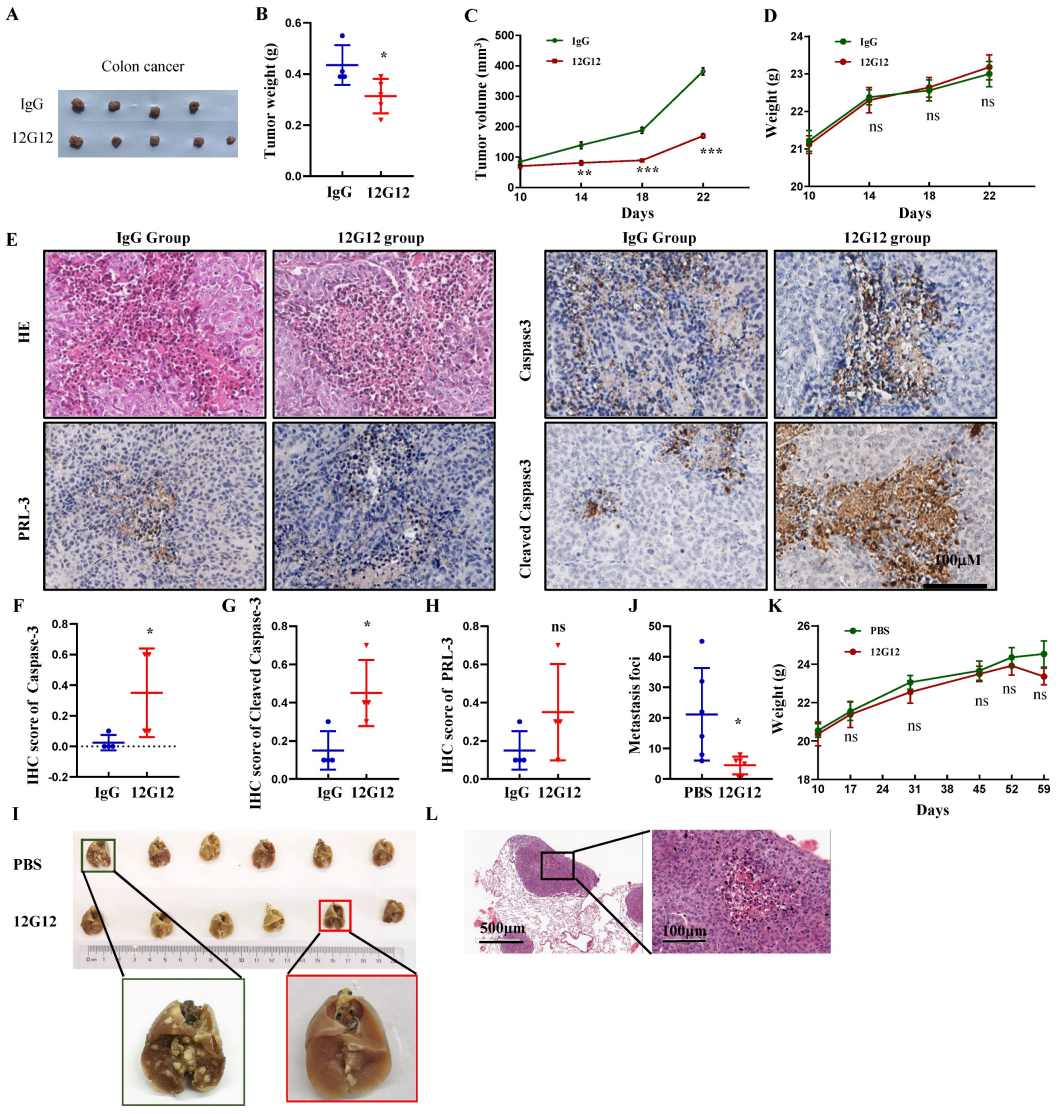
** mAb 12G12 inhibited colon cancer tumor growth and metastasis to lung in mouse models. A-G** Effects of mAb 12G12 on the tumor growth in Balb/c-nude mice. The mice were intravenously injected with antibody (10 mg/kg) or 40 mg/kg control IgG twice a week for 4 weeks at 5 day after intravenous injection of 5×10^6^ LoVo cells. All mice were euthanized and harvested. **A.** Representative photographs of tumors. **B and C.** Average tumor weights (**B**) and volume (**C**) monitored every 4 days for four times. **D.** Animal body weight monitored on the indicated days. **E.** HE staining and representative images of PRL-3, caspase-3 and cleaved caspase-3 staining in tumor tissues. Magnification, 200 ×. Bar, 100 μm. **F-H**. Quantitative IHC score of caspase-3 (**F**), cleaved caspase-3 (**G**) and PRL-3 (**H**).** I-L.** Effect of the mAb12G12 on the metastasis of LoVo cells in Balb/c-nude mice. 5×10^5^ LoVo cells were injected via vein tail, 12G12 antibody (20 mg/kg) and PBS was subsequently intravenously injected twice a week for 3 weeks. All mice were euthanized at 35 day. **I.** Representative photographs of tumors. **J.** Average metastasis loci in lung tissue. **K.** Animal body weight monitored on indicated days. **L**. HE staining of mice lung tissue. Bar, 100 μm. *, *p* <0.05; ***, *p* <0.001.

To assess the application of mAb 12G12 in vivo, we developed xenograft Subcutaneous tumor model implanted with LoVo cells (Figure 4A). mAb 12G12 dramatically decreased the tumor weight (0.44 ± 0.06 g VS 0.31 ± 0.09 g, *p* = 0.04) and volume compared to IgG control (Figure 4B and 4C). We didn't observe the loss of body weight in all the groups (*p* = 0.577, Figure 4D). HE staining confirmed the malignant cell assembles in situ (Figure 4E). Representative images and quantitative score indicated that caspase-3 and cleaved caspase-3 were higher in mAb 12G12 groups than in IgG group (caspase-3: 0.35 ± 0.14 VS 0.025 ± 0.05, *p* = 0.024; cleaved caspase-3: 0.45 ± 0.087 VS 0.15 ± 0.05, *p* = 0.032). PRL-3 expression was found in all the groups and there was not significant difference between IgG group and 12G12 group (0.15 ± 0.05 VS 0.35 ± 0.13, *p* = 0.190) (Figure 4E-4H).

Lung metastasis is frequently observed in colon cancer and associated with poor prognosis. Consistent with the in vitro results, 12G12 reduced the metastatic foci on lung compared to PBS control (4.50 ± 2.88 VS 21.17 ± 15.10, p = 0.024) (Figure 4I and 4J). There was no significant change of body weight (Figure 4K). Metastasis foci in the lung was also confirmed by the HE staining (Figure 4L).

### mAb 12G12 inhibited tumor growth in patient derived xenograft (PDX) model

We further investigated the efficacy of mAb 12G12 in gastric cancer PDX model. The detail clinical pathological information of patients was listed in the Supplementary Table 1. mAb 12G12 dramatically inhibited the tumor growth in all three PDX models (Supplementary Figure 4 A and 4B). PRL-3 level in tissue sections was determined by IHC and the presence of malignant cells in situ was confirmed by HE staining. Levels of caspase-3 and cleaved caspase-3 were significantly increased in mAb 12G12-treated groups (Supplementary Figure 4C). Taken the results of xenograft subcutaneous tumor model and PDX model together, mAb 12G12-suppressed tumor growth was associated with the triggering of apoptosis.

### mAb 12G12 inhibited cell migration in a TMED10-dependent manner

**Figure 5:**
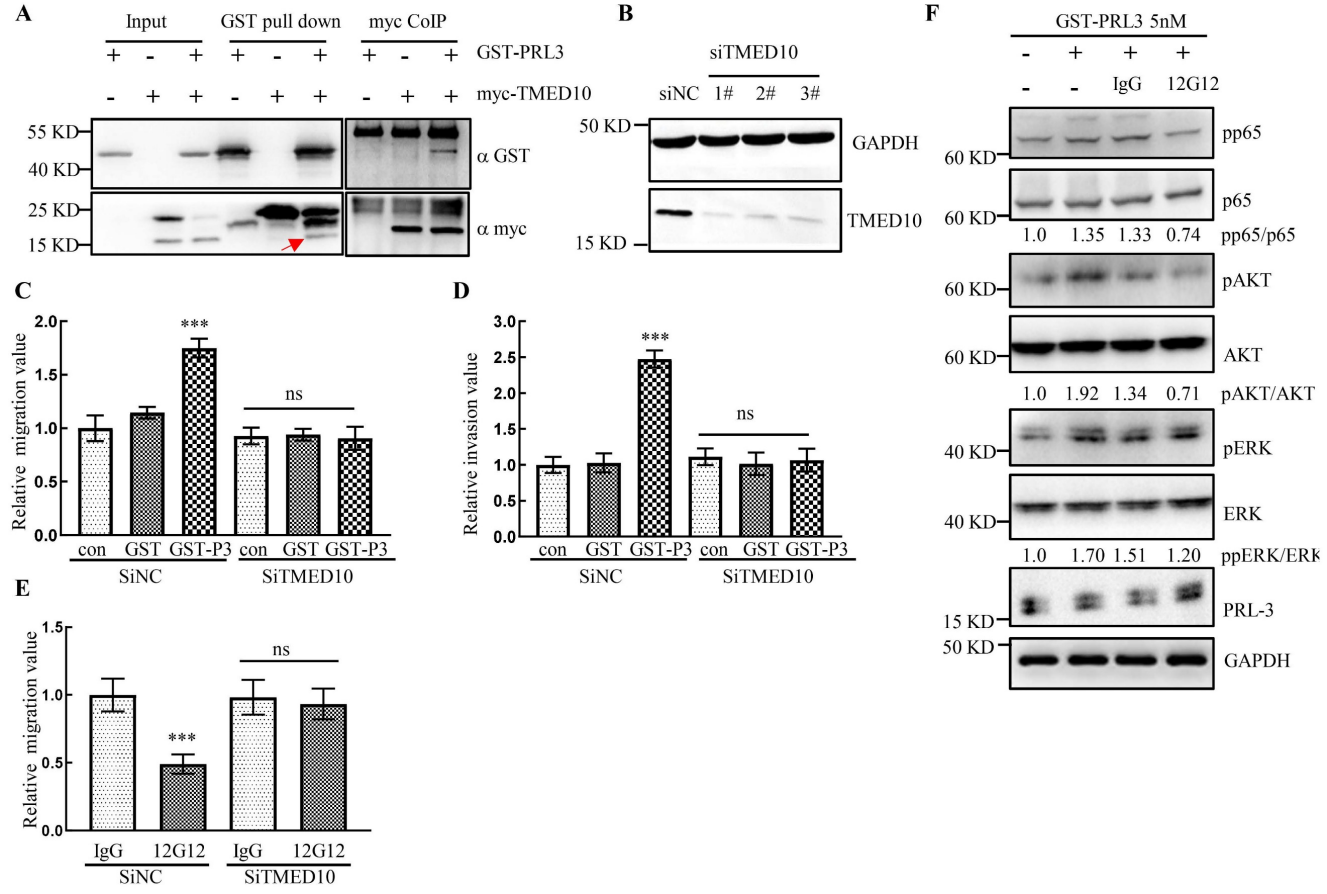
** mAb 12G12 inhibited cell migration in a TMED10-dependent manner. A.** Interaction between GST-PRL-3 and myc-TMED10 detected by co-immunoprecipitation and GST pull-down. Red arrow, endogenous TMED10**. B.** Western blotting analysis of TMED10 knockdown in HCT116 cells. **C, D.** Knockdown of TMED10 counteracted PRL-3 protein-promoted migration (**C**) and invasion (**D**). **E.** Knockdown of TMED10 abolished mAbs 12G12-inhibited migration.** F.** Levels of PRL-3-related signaling proteins in the HCT116 cells under treatment with GST-PRL-3 plus mAb 12G12. Quantification were measured and calculated by Image J. ***, *p* <0.001.

It has been reported that PRL-3 protein could “inside-out” externalize on the cell surface and exosomal membrane 15, however the mechanism remains elusive. Our previous study reported that PRL-3 localized in cytoplasm and nucleus by interacting with telomere protein RAP1 10, 21. Yeast two-hybrid screening identified a panel of PRL-3 partners 21, 22, one of which was identified as the Golgi protein TMED10 (Transmembrane p24 trafficking protein 10 Gene). CoIP and GST pull-down experiments showed that PRL-3 interacted with TMED10 (Figure 5A). Knockdown of TMED10 expression by siRNA was confirmed by Western blotting (Figure 5B). In the subsequent experiments we combined 3 knockdown siRNAs together to maximize the inhibition of TMED10 expression. We found that exogenously purified GST-PRL-3 could promote cell migration and invasion (Figure 5C and 5D). After silencing TMED10 expression by siRNA, GST-PRL-3-promoted migration and invasion were abolished (Figure 5C and 5D). Similarly, mAb 12G12-inhibited cell migration was abrogated (Figure 5E). It indicated that the effects of PRL-3 protein or mAb 12G12 on cell motility could be TMED10-dependent. Next, we detected the levels of PRL-3-related signaling proteins in the cells under treatment with GST-PRL-3 plus mAb 12G12 (Figure 5F). We found that showed that 12G12 was able to inhibit the phosphorylation of p65, AKT and ERK, which was activated by PRL-3 protein (Figure 5F). These results were consistent with the role of PRL-3 in activating diverse signaling pathways 23. mAb 12G12 suppressed tumor in a TMED10 dependent and through NFκB-AKT pathway.

## Discussion

The role of PRL-3 has been concerned for almost 20 years and its role in tumor initiation and metastasis has been demonstrated in both our previous study and by other groups 5, 10, 23. PRL-3 promoted tumor progression through various pathways, such as PI3K-AKT-ERK, NFκB and Jak2-STATs pathways 21, 24-26. Recent researches showed that PRL-3 also participated in the regulation of tumor microenvironment via recruiting B cell, NK cells and macrophages 16. Our previous study confirmed that DNA vaccine with plasmid encoding PRL-3 triggered CTL and T helper type 1 immune response 18. These studies revealed that targeting PRL-3 is not only hopeful in monoclonal antibody therapy but also in adjuvant therapy with immune inhibitions.

Using different immunogens, including prokaryotic protein, prokaryotic plasmids and peptides, we generated 13 different mAbs targeting PRL proteins. Through antibody specificity identification, we got 3 antibodies which specifically recognized PRL-3. mAb 12G12 is the “orphan” antibody probably targets the phosphorylation domain of PRL-3, which was different from binding domain of commercial clone 318 (Figure 1D). We tried to connect the anti-metastasis (Supplementary Figure 2) and anti-proliferation efficiency (Supplementary Figure 3) of mAb 12G12 to the endogenous PRL-3 status in tumor cells. Unfortunately, mAb 12G12 consistently inhibited the cell migration and invasion of colon cancer, gastric cancer and esophagus cancer independent of PRL-3 expression. For example, compared to HCT116, LoVo cells have lower PRL-3 expression, but mAb 12G12 has higher suppression of cell migration. Furthermore, the inhibitory efficiency of mAb 12G12 was negatively correlated with PRL-3 expression in lung cancer (lower expression of PRL-3 in PG, higher suppression of cell migration) and pancreatic cancer (higher expression of PRL-3 in ASPC-1, lower suppression of cell migration) (Supplementary Figure 2B-2G). Our previous study and other group's reports indicated that the prospect of PRL-3 research may hide in the tumor microenvironment, including secreting factors, exosomes, and immune cells 15, 25, 27. Therefore, we believe that mAb 12G12 may be applied in the treatment of solid tumors through different mechanisms. Besides 12G12, mAbs 8B7 and D3 also suppressed the cell migration and/or invasion in colon cancer cell lines (Figure 2). As we all know, preclinic antitumor drug development is full of uncertain factors. Our study potentially increased the success rate of developing antitumor mAbs to PRL-3.

Other group reported that antibody targeting PRL-3 eliminated PRL-3+ liver tumors and gastric tumors in vivo 15, 16. In our study, mAb 12G12 suppressed the tumor growth of colon cancer cell-derived and gastric cancer tissue-derived mice model. 12G12 also inhibited lung metastasis of colon cancer cells (Figure 4 and Supplementary Figure 4). Levels of caspase-3 and cleaved caspase-3 were elevated upon mAb 12G12 treatment (Figure 4E-4G and Supplementary Figure 4C), indicating that mAb 12G12 suppressed the tumor growth by triggering apoptosis pathways. Notably, 12G12 had negligible effect on body weight of mice in the in vivo experiments, suggesting the safety of using this mAb.

PRL-3 zumab, a hopeful antitumor drug, could be targeted to exosomal membrane and trigger the ADCC/ADCP cascade. Our previous study showed that PRL-3 protein translocates from cytoplasm to nuclei, even telomere end by forming a PRL-3-RAP1-TRF2 complex 10. We found that TMED10, a trafficking protein localized on the cell membrane, could directly interact with PRL-3 (Figure 5A). Knockdown of TMED10 not only counteracted PRL-3-promoted migration and invasion, but also blocked the 12G12's inhibitory effect on cell migration (Figure 5C-5E). It was revealed that TMED10 functions as a protein channel for vesicle entry and secretion. TMED10 also regulated the cytokine IL1β secretion 28, 29. There is also a cross-talk between cytokines and PRL-3 expression on tumors. Overexpression of PRL-3 induced the secretion of IL1α in a NF-κB-JAK-STAT3 dependent manner. Inhibition of IL1α level suppressed the PRL-3-promoted cell migration 23. Therefore, we supposed that TMED10 may mediate translocation of PRL-3 to plasma membrane, thereby facilitating recognition by mAb 12G12.

Collectively, our results confirmed the antitumor activity of mAb 12G12 in colorectal adenocarcinoma. Considering the role of PRL-3 in tumor immune microenvironment and the success of immunotherapy in clinics, mAb 12G12 combine with immune inhibitor, chemotherapy or radiotherapy should also be evaluated in the future.

## Supplementary Material

Supplementary figures.

## References

[1] Herbst RS, Morgensztern D, Boshoff C (2018). The biology and management of non-small cell lung cancer. Nature.

[2] Alexander M, Kim SY, Cheng H (2020). Update 2020: Management of Non-Small Cell Lung Cancer. Lung.

[3] Kitazawa M, Miyagawa Y, Koyama M, Nakamura S, Hondo N, Miyazaki S (2021). Drug sensitivity profile of minor KRAS mutations in colorectal cancer using mix culture assay: The effect of AMG-510, a novel KRAS G12C selective inhibitor, on colon cancer cells is markedly enhanced by the combined inhibition of MEK and BCL-XL. Molecular and clinical oncology.

[4] Kaiser J (2019). After decades, progress against an 'undruggable' cancer target. Science (New York, NY).

[5] Duciel L, Monraz Gomez LC, Kondratova M, Kuperstein I, Saule S (2019). The Phosphatase PRL-3 Is Involved in Key Steps of Cancer Metastasis. Journal of molecular biology.

[6] Xing X, Peng L, Qu L, Ren T, Dong B, Su X (2009). Prognostic value of PRL-3 overexpression in early stages of colonic cancer. Histopathology.

[7] Dai N, Lu AP, Shou CC, Li JY (2009). Expression of phosphatase regenerating liver 3 is an independent prognostic indicator for gastric cancer. World journal of gastroenterology.

[8] Ren T, Jiang B, Xing X, Dong B, Peng L, Meng L (2009). Prognostic significance of phosphatase of regenerating liver-3 expression in ovarian cancer. Pathology oncology research: POR.

[9] Wang L, Peng L, Dong B, Kong L, Meng L, Yan L (2006). Overexpression of phosphatase of regenerating liver-3 in breast cancer: association with a poor clinical outcome. Annals of oncology: official journal of the European Society for Medical Oncology.

[10] Lian S, Meng L, Yang Y, Ma T, Xing X, Feng Q (2017). PRL-3 promotes telomere deprotection and chromosomal instability. Nucleic acids research.

[11] Zhang C, Qu L, Lian S, Meng L, Min L, Liu J (2019). PRL-3 Promotes Ubiquitination and Degradation of AURKA and Colorectal Cancer Progression via Dephosphorylation of FZR1. Cancer research.

[12] Garcia EG, Veloso A, Oliveira ML, Allen JR, Loontiens S, Brunson D (2021). PRL3 enhances T-cell acute lymphoblastic leukemia growth through suppressing T-cell signaling pathways and apoptosis. Leukemia.

[13] Wei M, Haney MG, Rivas DR, Blackburn JS (2020). Protein tyrosine phosphatase 4A3 (PTP4A3/PRL-3) drives migration and progression of T-cell acute lymphoblastic leukemia in vitro and in vivo. Oncogenesis.

[14] Chong PSY, Chooi JY, Lim JSL, Toh SHM, Tan TZ, Chng WJ (2021). SMARCA2 is a novel interactor of NSD2 and regulates pro-metastatic PTP4A3 through chromatin remodeling in t(4;14) multiple myeloma. Cancer research.

[15] Thura M, Al-Aidaroos AQ, Gupta A, Chee CE, Lee SC, Hui KM (2019). PRL3-zumab as an immunotherapy to inhibit tumors expressing PRL3 oncoprotein. Nature communications.

[16] Thura M, Al-Aidaroos AQO, Yong WP, Kono K, Gupta A, Lin YB (2016). PRL3-zumab, a first-in-class humanized antibody for cancer therapy. JCI insight.

[17] Thura M, Ye Z, Al-Aidaroos AQ, Xiong Q, Ong JY, Gupta A (2021). PRL3 induces polypoid giant cancer cells eliminated by PRL3-zumab to reduce tumor relapse. Communications biology.

[18] Lv J, Liu C, Huang H, Meng L, Jiang B, Cao Y (2013). Suppression of breast tumor growth by DNA vaccination against phosphatase of regenerating liver 3. Gene therapy.

[19] Hardy S, Kostantin E, Hatzihristidis T, Zolotarov Y, Uetani N, Tremblay ML (2018). Physiological and oncogenic roles of the PRL phosphatases. The FEBS journal.

[20] Fiordalisi JJ, Dewar BJ, Graves LM, Madigan JP, Cox AD (2013). Src-mediated phosphorylation of the tyrosine phosphatase PRL-3 is required for PRL-3 promotion of Rho activation, motility and invasion. PLoS One.

[21] Lian S, Meng L, Liu C, Xing X, Song Q, Dong B (2013). PRL-3 activates NF-κB signaling pathway by interacting with RAP1. Biochemical and biophysical research communications.

[22] Peng L, Jin G, Wang L, Guo J, Meng L, Shou C (2006). Identification of integrin alpha1 as an interacting protein of protein tyrosine phosphatase PRL-3. Biochemical and biophysical research communications.

[23] Yang Y, Lian S, Meng L, Qu L, Shou C (2017). Antibody Array Revealed PRL-3 Affects Protein Phosphorylation and Cytokine Secretion. PloS one.

[24] Wang H, Quah SY, Dong JM, Manser E, Tang JP, Zeng Q (2007). PRL-3 down-regulates PTEN expression and signals through PI3K to promote epithelial-mesenchymal transition. Cancer research.

[25] Kozlov G, Funato Y, Chen YS, Zhang Z, Illes K, Miki H (2020). PRL3 pseudophosphatase activity is necessary and sufficient to promote metastatic growth. The Journal of biological chemistry.

[26] Chong PSY, Zhou J, Lim JSL, Hee YT, Chooi JY, Chung TH (2019). IL6 Promotes a STAT3-PRL3 Feedforward Loop via SHP2 Repression in Multiple Myeloma. Cancer research.

[27] Song Q, Zheng Y, Wu J, Wang S, Meng L, Yao Q (2021). PTP4A3 Is a Prognostic Biomarker Correlated With Immune Infiltrates in Papillary Renal Cell Carcinoma. Frontiers in immunology.

[28] Anwar MU, Sergeeva OA, Abrami L, Mesquita FS, Lukonin I, Amen T (2022). ER-Golgi-localized proteins TMED2 and TMED10 control the formation of plasma membrane lipid nanodomains. Developmental cell.

[29] Zhang M, Liu L, Lin X, Wang Y, Li Y, Guo Q (2020). A Translocation Pathway for Vesicle-Mediated Unconventional Protein Secretion. Cell.

