# Supplementary material for: Anti-PRL-3 Monoclonal Antibody inhibits the Growth and Metastasis of colorectal adenocarcinoma
Source: J Cancer. 2023 Aug 21;14(13):2585–95. doi: 10.7150/jca.81702 (PMC10475362; doi:10.7150/jca.81702)
Supplement: Supplementary file 1 — Supplementary figures. [file jcav14p2585s1.pdf]

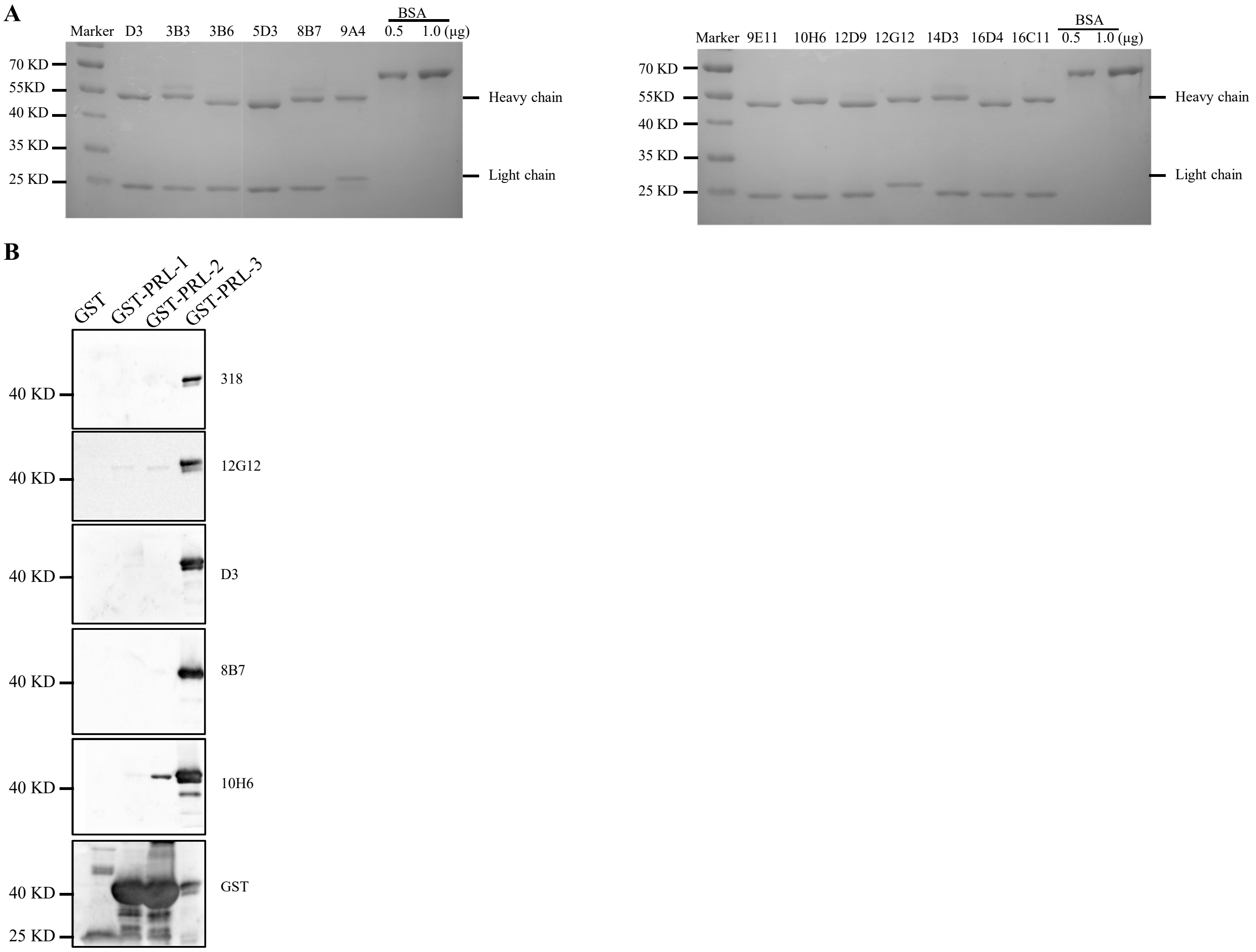

Supplemental Figure 1. Specificity identification of anti-PRLs antibodies. A. Coomassie brilliant blue staining of anti-PRLs antibodies. B. 10 times GST-PRL-1(5.0 µg), GST-PRL-2 (5.0 µg) and 1-time GST-PRL-3 (0.5µg) was loaded on SDS-PAGE gel to identify the specificity of PRL-3 mAbs.

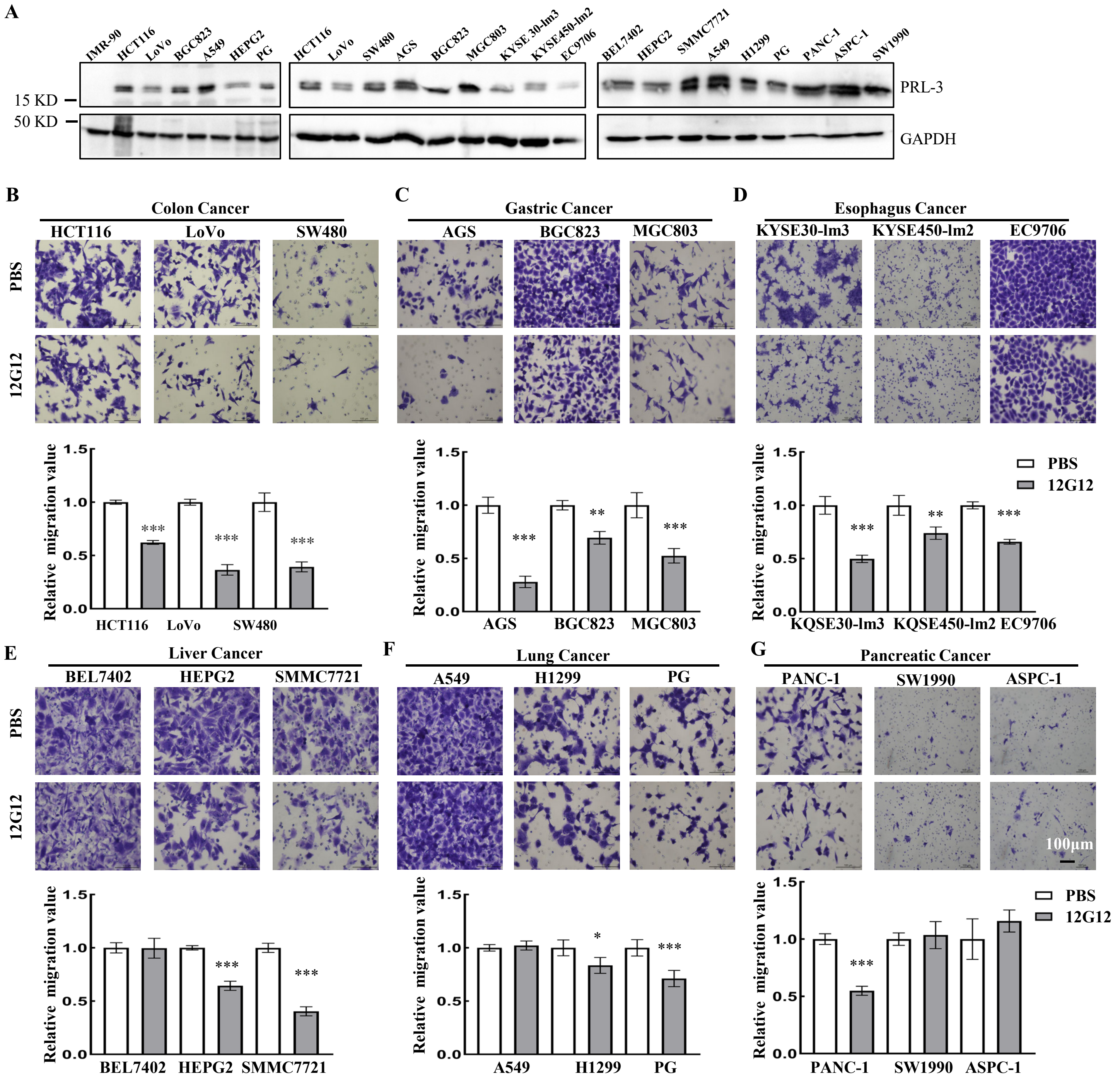

Supplemental Figure 2. Effect of the anti-PRL3 antibodies on the motility of colon cancer cells. A. Expression of endogenous PRL-3 in human fibroblast IMR-90, colon cancer, gastric cancer, esophagus cancer, liver cancer, lung cancer and pancreatic cancer cell lines. Transwell migration assay of colon cancer (B), gastric cancer (C), esophagus cancer (D), liver cancer (E), lung cancer (F) and pancreatic cancer (G) with mAb 12G12 and PBS treatment. The experiments were repeated three times. The values were the mean and standard deviation of normalize the number of each group with PBS (*P<0.05, **P<0.01, ***P<0.001).

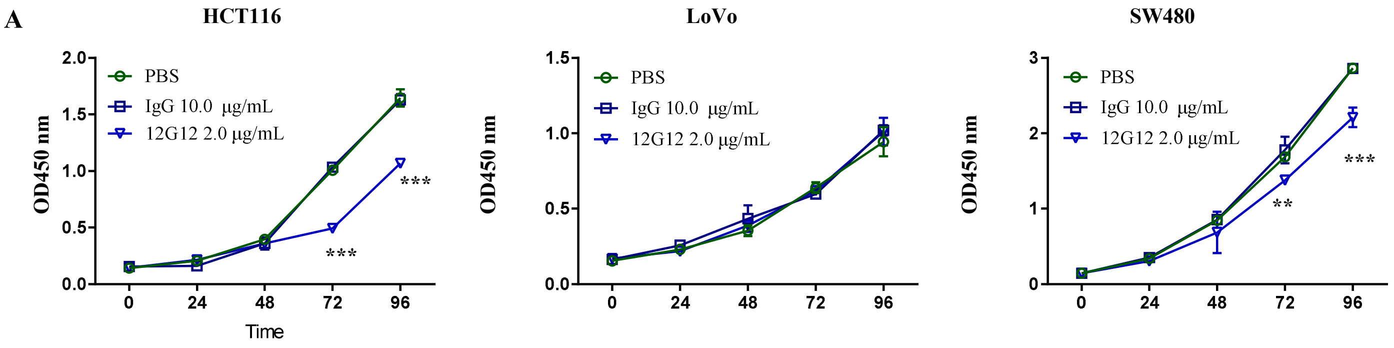

Supplemental Figure3. Effect of the anti-PRL3 antibodies on the proliferation of colon cancer. Cell proliferation of HCT116, LoVo and SW480 cells with 12G12 treatment (2.0μg/ml). PBS and IgG (10.0 μg/ml) treatment were set as controls. The experiments were repeated three times. The values are the mean and standard deviation of normalize the number of each group with PBS. (**P<0.01, ***P<0.001).

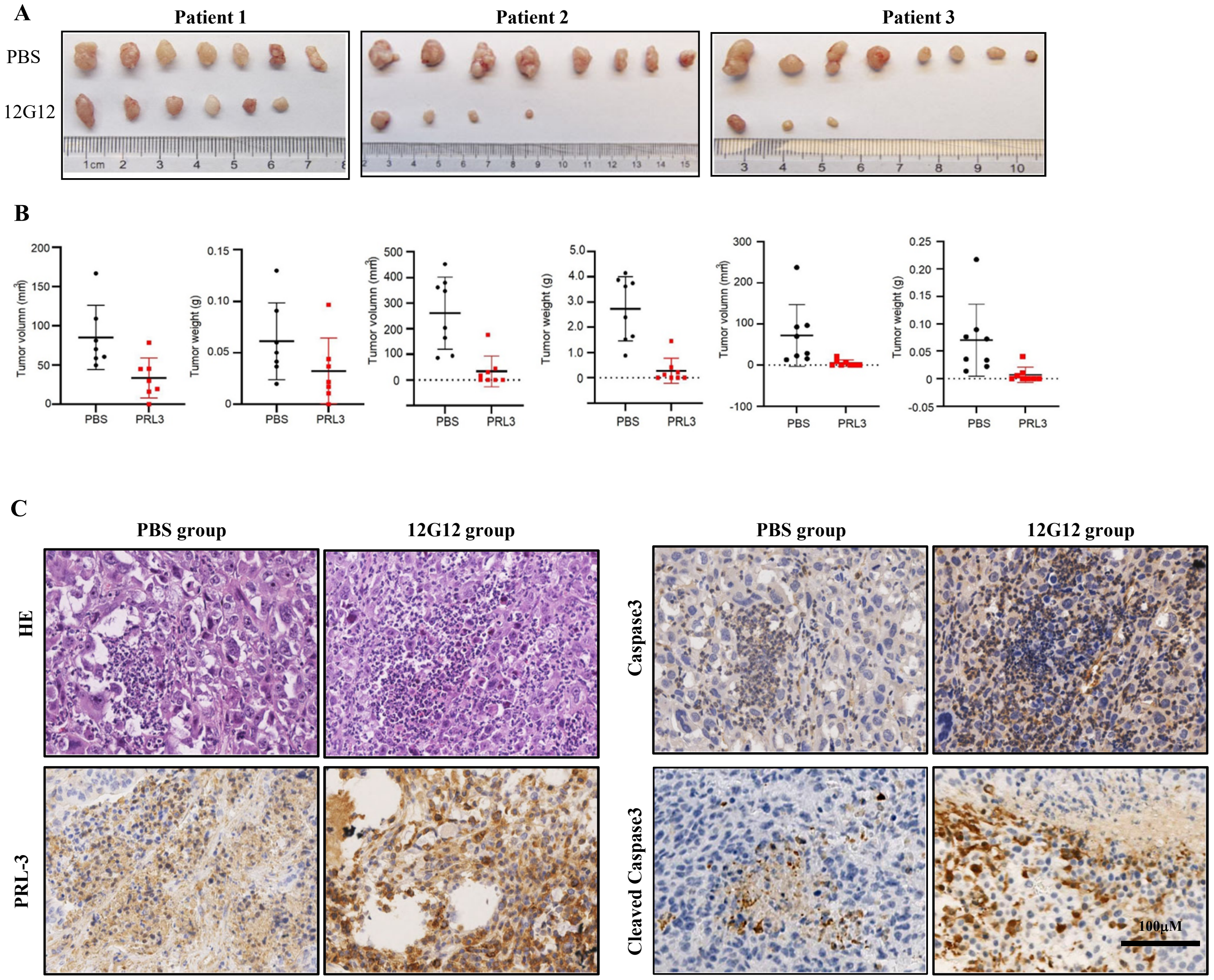

Supplemental Figure 4. In vivo efficacy study of mAb 12G12 in PDX model. Gastric cancer tissues from patients were injected into 16 Balb/c-nude mice. mAb 12G12 antibody (10 mg/kg) and PBS were administrated twice a week on day 3 after tumor inoculation. All mice were euthanized and harvest tumor samples. A. Representative photographs of tumors were shown. B. Average tumor weights and volumes. C. HE staining and representative images of PRL-3, caspase-3 and cleaved caspase-3 in tumor tissues of different groups. Magnification, 200 ×. Bar, 100 μM.
